# Factors associated with severe lower extremity artery disease in type 2 diabetes based on a large scale claims database in Japan

**DOI:** 10.1038/s41598-025-03797-9

**Published:** 2025-06-03

**Authors:** Takeshi Horii, Marina Kawaguchi, Yuichi Ikegami, Yoichi Oikawa, Akira Shimada, Kiyoshi Mihara

**Affiliations:** 1 Department of Pharmacy, Faculty of Pharmacy, Musashino University, Tokyo, Japan; 2 Department of Endocrinology and Diabetes, School of Medicine, Saitama Medical University, Saitama, Japan

**Keywords:** Diabetic foot lesions, Type 2 diabetes, Lower extremity artery disease, Revascularization, Endocrinology, Endocrine system and metabolic diseases, Diabetes

## Abstract

Diabetic foot lesions are becoming increasingly prevalent due to increases in type 2 diabetes (T2D), aging populations, and atherosclerotic diseases. Individuals with T2D with comorbid lower extremity artery disease (LEAD) are particularly susceptible. Although pharmacological therapies are often combined with risk management strategies, like exercise and smoking cessation, their roles in preventing LEAD progression are unclear. Herein, we investigated factors suppressing LEAD progression in T2D. Using data from the DeSC database, this retrospective cross-sectional study defined severe LEAD as a diagnosis requiring revascularization (Revasc). Logistic regression analysis was used to calculate odds ratios (ORs) for associated factors. The analysis included 243,606 patients with T2D divided into two groups; those with (*n* = 890) and without (*n* = 242,716) Revasc. Subgroup analysis of patients with LEAD (*n* = 27,258) with (*n* = 890) and without (*n* = 26,368) Revasc was also conducted. Sodium-glucose cotransporter 2 inhibitors (SGLT2is) (OR 0.50), metformin (OR 0.78), and fibrates (OR 0.75) were associated with a significant reduction in severe LEAD in the primary and subgroup analyses. In conclusion, the active use of SGLT2 inhibitors, metformin, and fibrates may help prevent LEAD progression. However, these medications are associated with adverse events, making it essential to manage patients individually to optimize benefits while minimizing risks.

**Supplementary Information:**

The online version contains supplementary material available at 10.1038/s41598-025-03797-9.

## Introduction

Type 2 diabetes (T2D) is associated with both microvascular and macrovascular complications due to persistent hyperglycemia^1–3^ Among these, diabetic foot lesions have become increasingly prevalent due to the increase in T2D incidence, aging populations, and atherosclerotic diseases. Individuals with T2D are particularly susceptible to developing foot lesions when they have comorbid lower extremity artery disease (LEAD)^4^. Systematic reviews^5^ on LEAD have identified diabetes as a significant risk factor for its onset. According to a survey by Japan’s Ministry of Health, Labour and Welfare, 0.7% of patients with diabetes have foot gangrene^6^. Internationally, the prevalence of diabetic foot ulcers ranges from 1.5 to 10%, with an incidence of 2.2–5.9%^7–9^. Meta-analyses^10^ in Japan have demonstrated that diabetes impacts LEAD more significantly than coronary artery disease (CAD). In a study focusing on patients with T2D with a mean age of 61 years, the prevalence of an ankle-brachial index (ABI) below 0.90 was reported to be 7.0%. Among patients aged 65 years or older, this prevalence increased to 12.7%^11^. Moreover, patients with T2D and an ABI between 0.90 and 1.00 exhibit a high risk of progressing to LEAD, necessitating careful longitudinal monitoring^12^. Hypertension and dyslipidemia, common risk factors for T2D, are also shared risk factors for LEAD. The overlapping of these risk factors results in a 1.5- to 10-fold increase in the incidence of LEAD^13^. Additionally, LEAD frequently coexists with other atherosclerotic conditions, such as CAD and cerebrovascular disease (CVD), with adjusted analyses indicating a 1.5- to 2-fold higher risk of cardiovascular events in patients with LEAD^14^.

The treatment strategy for LEAD varies significantly depending on the severity of symptoms and degree of ischemia. In cases of chronic limb-threatening ischemia (CLTI), characterized by infection or critical ischemia, surgical interventions such as debridement, minor amputations, or revascularization (Revasc) procedures are frequently necessitated. The risk of symptom recurrence within 3 years following Revasc has been reported to be 30% for bypass surgery and 64% for endovascular therapy (EVT)^15^. Regarding prognosis, cumulative mortality rates post-Revasc are approximately between 10% and 15% at 3 years^15–17^, while the 5-year mortality following EVT approaches 20%^18,19^. In advanced cases, major amputations aimed at alleviating ischemic pain and removing necrotic or infected tissues become necessary. Although efficacious, major amputations significantly impact patients’ quality of life. Moreover, additional procedures, including debridement or re-amputation, are required in 4–40% of cases depending on the level of amputation^20–22^.

Rehospitalization rates following toe or foot amputations are approximately 20%, with the majority occurring within the first month^20–22^. The prognosis following major amputations in patients with diabetes and CLTI is less favorable than that in non-diabetic patients, with the cumulative survival rates at 1, 3, 5, and 10 years reported to be 78%, 61%, 44%, and 19%, respectively^23^. Comprehensive risk factor management is essential not only for preventing the onset and progression of LEAD, but also for improving patient outcomes. Despite this, LEAD is often underdiagnosed in clinical practice, and even when diagnosed, is frequently inadequately managed^24–27^. While pharmacological therapies are frequently combined with comprehensive risk management strategies, such as exercise and smoking cessation, there is a paucity of reports that adequately examine the contribution of these interventions in preventing the progression of LEAD.

Given that patients with advanced LEAD and type 2 diabetes (T2D) often present with multiple comorbidities, a comprehensive evaluation of concomitant medications and comorbid conditions is necessary to assess their potential impact. To address this knowledge gap, we conducted a large-scale retrospective cross-sectional study utilizing a healthcare claims database encompassing the working-age population in Japan, with the aim of investigating factors inhibiting the progression of LEAD in patients with T2D.

## Results

Table 1 shows baseline patient characteristics. The mean age was 61.9 ± 8.4 years, with a nearly equal distribution of men and women. Approximately 3% of patients were classified as underweight (BMI < 18.5 kg/m²). Revasc was confirmed in 890 of 243,606 patients (0.37%). There were significant differences in many clinical parameters between the Revasc (+) and Revasc (−) groups. Compared with the Revasc (-) group, the Revasc (+) group had a larger proportion of men, were older, and had a lower BMI. The frequency of complications and concomitant medication use in each group are shown in Table 1.

**Table 1:** Baseline characteristics of patients with type 2 diabetes classified by the presence or absence of revascularization.

	Characteristics	Overall (*n* = 243,606)	Revasc (−) groups (*n* = 242,716)	Revasc (+) groups (*n* = 890)	*P* value
Sex, n (%)	Male	139,525 (57.3)	138,787 (57.2)	738 (82.9)	< 0.001
	Female	104,081 (42.7)	103,929 (42.8)	152 (17.1)	
Age	Mean (years)	61.9 ± 8.4	61.8 ± 8.4	64.3 ± 0.5	< 0.001
BMI	Mean (kg/m^2^)	25.2 ± 4.2	25.2 ± 4.2	24.3 ± 3.7	0.028
	Distribution, n (%)				
	≥ 18.5	236,616 (97.1)	235,761 (97.1)	855 (96.1)	< 0.001
	< 18.5	6990 (2.9)	6955 (2.9)	35 (3.9)	
HbA1c	Mean (%)	6.7 ± 1.4	6.7 ± 1.4	7.0 ± 1.6	< 0.001
	Distribution, n (%)				
	≥ 7.0	78,733 (32.3)	78,369 (32.3)	364 (40.9)	< 0.001
	< 7.0	164,873 (67.7)	164,347 (67.7)	526 (59.1)	
eGFR	Mean (mL/min/1.73m^2^)	74.0 ± 17.8	74.0 ± 17.8	63.9 ± 23.6	< 0.001
	Distribution, n (%)				
	≥ 60	198,716 (81.6)	198,161 (81.6)	555 (62.4)	< 0.001
	30 to <60	42,917 (17.6)	42,657 (17.6)	260 (29.2)	
	< 30	1973 (0.8)	1898 (0.8)	75 (8.4)	
Diastolic blood pressure	Mean (mmHg)	82.2 ± 11.9	82.2 ± 11.9	81.0 ± 12.4	0.001
	Distribution, n (%)				
	≥ 80	145,544 (59.7)	145,069 (59.8)	475 (53.4)	< 0.001
	< 80	98,062 (40.3)	97,647 (40.2)	415 (46.6)	
Systolic blood pressure	Mean (mmHg)	139.7 ± 17.9	149.7 ± 17.8	145.8 ± 20.3	< 0.001
	Distribution, n (%)				
	≥ 130	175,913 (72.2)	175,210 (72.2)	703 (79.0)	< 0.001
	< 130	67,693 (27.8)	67,506 (27.8)	187 (21.0)	
HDL-C	Mean (mg/dL)	64.0 ± 17.8	64.0 ± 17.8	59.4 ± 17.2	< 0.001
	Distribution, n (%)				
	< 40	13,057 (5.4)	12,966 (5.3)	91 (10.2)	< 0.001
	≥ 40	230,549 (94.6)	229,750 (94.7)	799 (89.8)	
LDL-C	Mean (mg/dL)	131.0 ± 34.7	131.0 ± 34.7	122.3 ± 38.8	< 0.001
	Distribution, n (%)				
	≥ 120	149,354 (61.3)	148,901 (61.3)	453 (50.9)	< 0.001
	< 120	94,252 (38.7)	93,815 (38.7)	437 (49.1)	
TG	Mean (mg/dL)	177.9 ± 145.8	177.8 ± 145.7	200.6 ± 171.2	< 0.001
	Distribution, n (%)				
	≥ 150	114,646 (47.1)	114,172 (47.0)	474 (53.3)	< 0.001
	< 150	128,960 (52.9)	128,544 (53.0)	416 (46.7)	
Smoking history	No	208,807 (85.7)	208,170 (85.8)	637 (71.6)	< 0.001
	Yes	34,799 (14.3)	34,546 (14.2)	253 (28.4)	
Drinking history	Every day	45,174 (18.5)	44,950 (18.5)	224 (25.1)	< 0.001
	Sometimes	46,954 (19.3)	46,782 (19.3)	172 (19.3)	
	Rarely	141,484 (58.1)	141,043 (58.1)	441 (49.6)	
	No	9994 (4.1)	9941 (4.1)	53 (6.0)	
Complication					
Angina		51,079 (21.0)	50,594 (20.8)	485 (54.5)	< 0.001
Arrhythmia		45,369 (18.6)	45,047 (18.6)	322 (36.2)	< 0.001
Cerebral infraction		46,850 (19.2)	46,196 (19.0)	654 (73.5)	< 0.001
CKD		16,821 (6.9)	16,604 (6.8)	217 (24.4)	< 0.001
Heart failure		58,184 (23.9)	57,644 (23.7)	540 (60.7)	< 0.001
Hyperlipidemia		111,995 (46.0)	111,363 (45.9)	632 (71.0)	< 0.001
Hypertension		172,677 (70.9)	171,839 (70.8)	838 (94.2)	< 0.001
Myocardial infraction		12,215 (5.0)	1017 (0.4)	204 (22.9)	< 0.001
Neuropathy		84,930 (34.9)	84,465 (34.8)	465 (52.2)	< 0.001
Retinopathy		62,077 (25.5)	61,713 (25.4)	364 (40.9)	< 0.001
Anti-diabetic agent use	Distribution, n (%)				
DPP-4is		100,596 (41.3)	100,146 (41.3)	450 (50.6)	< 0.001
Glinides		13,674 (5.6)	13,595 (5.6)	79 (8.9)	< 0.001
GLP-1 receptor agonists		11,140 (4.6)	11,092 (4.6)	48 (5.4)	0.241
Imeglimin		1658 (0.7)	1655 (0.7)	3 (0.3)	0.212
Insulin		35,343 (14.5)	35,036 (14.4)	307 (34.5)	< 0.001
Metformin		74,907 (30.7)	74,633 (30.7)	274 (30.8)	0.981
SGLT2is		58,077 (23.8)	57,888 (23.9)	189 (21.2)	0.068
Sulfonylureas		30,071 (12.3)	29,896 (12.3)	175 (19.7)	< 0.001
Thiazolidinediones		16,575 (6.8)	16,501 (6.8)	74 (8.3)	0.073
α-GIs		25,175 (10.3)	25,030 (10.3)	145 (16.3)	< 0.001
Antiplatelet drug use	Distribution, n (%)				
Aspirin		30,570 (12.5)	30,121 (12.4)	449 (50.4)	< 0.001
P2Y12is		19,699 (8.1)	19,307 (8.0)	392 (44.0)	< 0.001
PDEis		6194 (2.5)	5929 (2.4)	265 (29.8)	< 0.001
Antihypertensive drug use	Distribution, n (%)				
ACEis		13,381 (5.5)	13,274 (5.5)	107 (12.0)	< 0.001
Aldosterone receptor blocker		12,658 (5.2)	12,518 (5.2)	140 (15.7)	< 0.001
ARBs		110,227 (45.2)	109,581 (45.1)	646 (72.6)	< 0.001
ARNI		3185 (1.3)	3140 (1.3)	45 (5.1)	< 0.001
Calcium channel blockers		126,088 (51.8)	125,352 (51.6)	736 (82.7)	< 0.001
Loop diuretics		20,370 (8.4)	20,126 (8.3)	244 (27.4)	< 0.001
Thiazide diuretics		18,369 (7.5)	18,214 (7.5)	155 (17.4)	< 0.001
V2-Receptor blockers		2227 (0.9)	180 (0.1)	42 (4.7)	< 0.001
α-Receptor blockers		7442 (3.1)	7325 (3.0)	117 (13.1)	< 0.001
β-Receptor blockers		45,346 (18.6)	44,888 (18.5)	458 (51.5)	< 0.001
Cholesterol-lowering drug use	Distribution, n (%)				
Ezetimibe		18,286 (7.5)	18,188 (7.5)	98 (11.0)	< 0.001
Eicosapentaenoic acid		15,019 (6.2)	14,913 (6.1)	106 (11.9)	< 0.001
Fibrates		23,294 (9.6)	23,214 (9.6)	80 (9.0)	0.560
PCSK9is		182 (0.07)	181 (0.1)	1 (0.1)	0.676
Statins		123,108 (50.5)	122,546 (50.5)	562 (63.1)	< 0.001

Data are presented as numbers (%) or means (standard deviations). The *P* value was calculated for the differences between patients with Revasc or without Revasc.

* Revasc* revascularization, *BMI* body mass index, *HbA1c* hemoglobin A1c, *eGFR* estimated glomerular filtration rate, *HDL-C* high-density lipoprotein cholesterol, *LDL-C* low-density lipoprotein cholesterol, *TG* triglycerides, *CKD* chronic kidney disease, *DPP-4is* dipeptidyl peptidase-4 inhibitors, *GLP-1* receptor agonists, glucagon-like peptide-1, *SGLT2is* sodium-glucose cotransporter 2 inhibitors, *α-GI* alpha-glucosidase inhibitor, *PDEis* phosphodiesterase inhibitors, *ACEis* angiotensin-converting enzyme inhibitors, *ARBs* angiotensin II receptor blockers, *ARNI* angiotensin receptor-neprilysin inhibitor, *V2-Receptor blockers* vasopressin V2 receptor blockers.

The risk of severe LEAD is presented in Table 2. Compared being a man, being a woman was associated with a lower OR for severe LEAD (OR 0.50; 95% CI 0.41–0.60). The OR for severe LEAD significantly increased per 10-year increments in age (OR 1.30; 95% CI 1.16–1.47). A BMI of less than 18.5 kg/m^2^, with a BMI of 18.5 kg/m^2^ or more as the reference, was also significantly associated with LEAD (OR 2.16; 95% CI 1.50–3.11). Moreover, an eGFR of less than 30 mL/min/1.73 m^2^ (OR 3.39; 95% CI 2.53–4.53) and between 30 and 60 mL/min/1.73 m^2^ (OR 1.33, 95% CI 1.14–1.55), with an eGFR of 60 mL/min/1.73 m^2^ or more as the reference, was significantly associated with LEAD. An HbA1c level of less than 7.0%, sBP, and HDL-C level of more than 40 mg/dL were associated with a significantly lower OR for severe LEAD when the target set by the guidelines was achieved. However, the OR for severe LEAD significantly increased when a dBp of less than 80 mg/dL was achieved. The OR increased in the presence of smoking habits, whereas alcohol consumption showed no association. The ORs for sodium-glucose cotransporter 2 inhibitors (SGLT2is), metformin, and fibrates were significantly lower than 1. In contrast, the use of insulin, antiplatelet drugs, and certain types of antihypertensive agents significantly increased the OR. Several SGLT2is and fibrates were investigated in detail (Supplementary Table S2). Among SGLT2is, dapagliflozin demonstrated a particularly low OR; however, all agents in this class tended to lower the OR. Fibrate use was associated with lower ORs for severe LEAD. Among fibrates, pemafibrate, which exerts anti-peroxisome proliferator-activated receptor alpha (PPARα) action, was associated with lower ORs for severe LEAD. Due to the limited number of patients who received clofibrate, it was not possible to analyze its effects.

**Table 2:** Logistic regression analysis of factors associated with the implementation of revascularization in patients with type 2 diabetes.

		Odds ratio	95% CI	*P* value
Sex	Male	Reference	–	
	Female	0.50	0.41–0.60	< 0.001
Age/10years		1.30	1.16–1.47	< 0.001
BMI (kg/m^2^)	≥ 18.5	Reference	–	
	< 18.5	2.16	1.50–3.11	< 0.001
HbA1c (%)	≥ 7.0	Reference	–	
	< 7.0	0.63	0.53–0.75	< 0.001
eGFR (mL/min/1.73m^2^)	≥ 60	Reference	–	
	30 to <60	1.33	1.14–1.55	< 0.001
	< 30	3.39	2.53–4.53	< 0.001
Diastolic blood pressure (mmHg)	≥ 80	Reference	–	
	< 80	1.54	1.32–1.80	< 0.001
Systolic blood pressure (mmHg)	≥ 130	Reference	–	
	< 130	0.83	0.69–0.99	0.048
HDL-C (mg/dL)	< 40	Reference	–	
	≥ 40	1.08	0.85–1.36	0.549
LDL-C (mg/dL)	≥ 120	Reference	–	
	< 120	0.95	0.83–1.10	0.526
TG (mg/dL)	≥ 150	Reference	–	
	< 150	0.88	0.76–1.01	0.077
Smoking history	No	Reference	–	
	Yes	2.05	1.75–2.40	< 0.001
Drinking history	Every day	Reference	–	
	Sometimes	1.05	0.85–1.29	0.667
	Rarely	0.90	0.76–1.08	0.268
	No	1.16	0.85–1.58	0.354
Anti-diabetic agent use				
DPP-4is		0.94	0.79–1.11	0.445
Glinides		0.92	0.71–1.20	0.537
GLP-1 receptor agonists		–	–	
Imeglimin		–	–	
Insulin		1.41	1.19–1.67	< 0.001
Metformin		0.78	0.65–0.92	0.005
SGLT2is		0.50	0.41–0.60	< 0.001
Sulfonylureas		1.21	0.99–1.47	0.059
Thiazolidinediones		0.98	0.76–1.27	0.903
α-GIs		1.11	0.90–1.36	0.332
Antiplatelet drug use				
Aspirin		1.99	1.67–2.36	< 0.001
P2Y12is		2.66	2.24–3.15	< 0.001
PDEis		7.15	6.11–8.38	< 0.001
Antihypertensive drug use				
ACEis		0.83	0.67–1.04	0.103
Aldosterone receptor blocker		1.21	0.96–1.51	0.104
ARBs		1.41	1.19–1.66	< 0.001
ARNI		1.41	1.01–1.96	0.042
Calcium channel blockers		1.97	1.62–2.39	< 0.001
Loop diuretics		1.29	1.06–1.57	0.01
Thiazide diuretics		1.18	0.98–1.43	0.082
V2-Receptor blockers		1.03	0.72–1.49	0.855
α-Receptor blockers		1.43	1.15–1.78	0.001
β-Receptor blockers		1.89	1.62–2.20	< 0.001
Cholesterol-lowering drug use				
Ezetimibe		0.85	0.68–1.07	0.166
Eicosapentaenoic acid		1.15	0.92–1.42	0.216
Fibrates		0.75	0.59–0.95	0.02
PCSK9is	–	–	–	
Statins		0.88	0.75–1.03	0.117

The data shows the results of the multivariate analysis. GLP-1 receptor agonist. Imeglimin, and PCSK9is were excluded from the univariate analysis.

*BMI* body mass index, *CI* confidence interval, *HbA1c* hemoglobin A1c, *eGFR* estimated glomerular filtration rate, *HDL-C* high-density lipoprotein cholesterol, *LDL-C* low-density lipoprotein cholesterol, *TG* triglycerides, *CKD* chronic kidney disease, *DPP-4is* dipeptidyl peptidase-4 inhibitors, *GLP-1* receptor agonists, glucagon-like peptide-1, *SGLT2is* sodium-glucose cotransporter 2 inhibitors, *α-GI* alpha-glucosidase inhibitor, *PDEis* phosphodiesterase inhibitors, *ACEis* angiotensin-converting enzyme inhibitors, *ARBs* angiotensin II receptor blockers, *ARNI* angiotensin receptor-neprilysin inhibitor, *V2-Receptor blockers* vasopressin V2 receptor blockers.

Next, the OR for severe LEAD was calculated exclusively for patients with T2D diagnosed with LEAD (Tables 3, 4). The patient background exhibited trends similar to those of patients with T2D without a diagnosis of LEAD. The OR for severe LEAD showed a similar trend, with SGLT2is and fibrates showing lower ORs. Additionally, the GLP-1 receptor agonists and ezetimibe exhibited significantly lower ORs. Finally, Supplementary Table S3 presents a detailed analysis of the SGLT2is and fibrates. Among the SGLT2is, all components showed low ORs, with dapagliflozin, empagliflozin, and ipragliflozin demonstrating statistically significant values. Among the fibrates, pemafibrate exhibited the lowest value.

**Table 3:** Baseline characteristics of patients with type 2 diabetes with lower extremity artery disease classified by the presence or absence of revascularization.

	Characteristics	Overall (*n* = 27,258)	Revasc (−) groups (*n* = 26,368)	Revasc (+) groups (*n* = 890)	*P* value
Sex, n (%)	Male	15,909 (58.4)	15,171 (57.5)	738 (82.9)	< 0.001
	Female	11,349 (41.6)	11,197 (42.5)	152 (17.1)	
Age	Mean (years)	62.5 ± 7.6	62.4 ± 7.6	64.3 ± 0.5	< 0.001
BMI	Mean (kg/m^2^)	25.1 ± 4.2	25.1 ± 4.2	24.3 ± 3.7	0.028
	Distribution, n (%)				
	≥ 18.5	26,355 (96.7)	25,500 (96.7)	855 (96.1)	< 0.001
	< 18.5	903 (3.3)	868 (3.3)	35 (3.9)	
HbA1c	Mean (%)	6.8 ± 1.6	6.8 ± 1.6	7.0 ± 1.6	< 0.001
	Distribution, n (%)				
	≥ 7.0	9585 (35.2)	9221 (35.0)	364 (40.9)	< 0.001
	< 7.0	17,673 (64.8)	17,147 (65.0)	526 (59.1)	
eGFR	Mean (mL/min/1.73m^2^)	72.6 ± 19.7	72.9 ± 19.4	63.9 ± 23.6	< 0.001
	Distribution, n (%)				
	≥ 60	21,509 (78.9)	20,954 (79.5)	555 (62.4)	< 0.001
	30 -<60	5216 (19.1)	4956 (18.8)	260 (29.2)	
	< 30	533 (2.0)	458 (1.7)	75 (8.4)	
Diastolic blood pressure	Mean (mmHg)	81.6 ± 12.6	81.6 ± 12.6	81.0 ± 12.4	0.001
	Distribution, n (%)				
	≥ 80	15,731 (57.7)	15,256 (57.9)	475 (53.4)	< 0.001
	< 80	11,527 (42.3)	11,112 (42.1)	415 (46.6)	
Systolic blood pressure	Mean (mmHg)	139.9 ± 18.4	139.7 ± 18.3	145.8 ± 20.3	< 0.001
	Distribution, n (%)				
	≥ 130	19,826 (72.7)	19,123 (72.5)	703 (79.0)	< 0.001
	< 130	7432 (27.3)	7245 (27.5)	187 (21.0)	
HDL-C	Mean (mg/dL)	64.3 ± 18.8	64.5 ± 18.9	59.4 ± 17.2	< 0.001
	Distribution, n (%)				
	< 40	1585 (5.8)	1494 (5.7)	91 (10.2)	< 0.001
	≥ 40	25,673 (94.2)	24,874 (94.3)	799 (89.8)	
LDL-C	Mean (mg/dL)	128.7 ± 35.8	129.0 ± 35.7	122.3 ± 38.8	< 0.001
	Distribution, n (%)				
	≥ 120	15,902 (58.3)	15,449 (58.6)	453 (50.9)	< 0.001
	< 120	11,356 (41.7)	10,919 (41.4)	437 (49.1)	
TG	Mean (mg/dL)	178.7 ± 147.2	178.0 ± 146.3	200.6 ± 171.2	< 0.001
	Distribution, n (%)				
	≥ 150	12,896 (47.3)	12,422 (47.1)	474 (53.3)	< 0.001
	< 150	14,362 (52.7)	13,946 (52.9)	416 (46.7)	
Smoking history	No	23,579 (86.5)	22,942 (87.0)	637 (71.6)	< 0.001
	Yes	3679 (13.5)	3426 (13.0)	253 (28.4)	
Drinking history	Every day	4714 (17.3)	4490 (17.0)	224 (25.1)	< 0.001
	Sometimes	4927 (18.1)	4755 (18.0)	172 (19.3)	
	Rarely	16,350 (60.0)	15,909 (60.3)	441 (49.6)	
	No	1267 (4.6)	1214 (4.7)	53 (6.0)	
Complication					
Angina		9654 (35.4)	9169 (34.8)	485 (54.5)	< 0.001
Arrhythmia		6919 (25.4)	6597 (25.0)	322 (36.2)	< 0.001
Cerebral infraction		8822 (32.4)	8168 (31.0)	654 (73.5)	< 0.001
CKD		2924 (10.7)	2707 (10.3)	217 (24.4)	< 0.001
Heart failure		9815 (36.0)	9275 (35.2)	540 (60.7)	< 0.001
Hyperlipidemia		14,693 (53.9)	14,061 (53.3)	632 (71.0)	< 0.001
Hypertension		21,199 (77.8)	20,361 (77.2)	838 (94.2)	< 0.001
Myocardial infraction		2402 (8.8)	2198 (8.3)	204 (22.9)	< 0.001
Neuropathy		13,758 (50.5)	13,293 (50.4)	465 (52.2)	< 0.001
Retinopathy		9758 (35.8)	9394 (35.6)	364 (40.9)	< 0.001
Anti-diabetic agent use	Distribution, n (%)				
DPP-4is		12,352 (45.3)	11,902 (45.1)	450 (50.6)	< 0.001
Glinides		2349 (8.6)	2270 (8.6)	79 (8.9)	< 0.001
GLP-1 receptor agonists		2160 (7.9)	2112 (8.0)	48 (5.4)	0.241
Imeglimin		237 (0.9)	234 (0.9)	3 (0.3)	0.212
Insulin		6356 (23.3)	6049 (22.9)	307 (34.5)	< 0.001
Metformin		9682 (35.5)	9408 (35.7)	274 (30.8)	0.981
SGLT2is		7624 (28.0)	7435 (28.2)	189 (21.2)	0.068
Sulfonylureas		4027 (14.8)	3852 (14.6)	175 (19.7)	< 0.001
Thiazolidinediones		2248 (8.2)	2174 (8.2)	74 (8.3)	0.073
α-GIs		3791 (13.9)	3646 (13.8)	145 (16.3)	< 0.001
Antiplatelet drug use	Distribution, n (%)				
Aspirin		6176 (22.7)	5727 (21.7)	449 (50.4)	< 0.001
P2Y12is		4434 (16.3)	4042 (15.3)	392 (44.0)	< 0.001
PDEis		1674 (6.1)	1409 (5.3)	265 (29.8)	< 0.001
Antihypertensive drug use	Distribution, n (%)				
ACEis		2011 (7.4)	1904 (7.2)	107 (12.0)	< 0.001
Aldosterone receptor blocker		2238 (8.2)	2098 (8.0)	140 (15.7)	< 0.001
ARBs		13,838 (50.8)	13,192 (50.0)	646 (72.6)	< 0.001
ARNI		581 (2.1)	536 (2.0)	45 (5.1)	< 0.001
Calcium channel blockers		15,440 (56.6)	14,704 (55.8)	736 (82.7)	< 0.001
Loop diuretics		3667 (13.5)	3423 (13.0)	244 (27.4)	< 0.001
Thiazide diuretics		2545 (9.3)	2390 (9.1)	155 (17.4)	< 0.001
V2-Receptor blockers		474 (1.7)	432 (1.6)	42 (4.7)	< 0.001
α-Receptor blockers		1245 (4.6)	1128 (4.3)	117 (13.1)	< 0.001
β-Receptor blockers		7709 (28.3)	7251 (27.5)	458 (51.5)	< 0.001
Cholesterol-lowering drug use	Distribution, n (%)				
Ezetimibe		2961 (10.9)	2863 (10.9)	98 (11.0)	< 0.001
Eicosapentaenoic acid		2741 (10.1)	2635 (10.0)	106 (11.9)	< 0.001
Fibrates		2825 (10.4)	2745 (10.4)	80 (9.0)	0.560
PCSK9is		51 (0.2)	50 (0.2)	1 (0.1)	0.676
Statins		15,899 (58.3)	15,337 (58.2)	562 (63.1)	< 0.001

Data are presented as numbers (%) or means (standard deviations). The *P* values were calculated for the differences between patients with Revasc or without Revasc.

* Revasc* revascularization, *BMI* body mass index, *HbA1c* hemoglobin A1c, *eGFR* estimated glomerular filtration rate, *HDL-C* high-density lipoprotein cholesterol, *LDL-C* low-density lipoprotein cholesterol, *TG* triglycerides, *CKD* chronic kidney disease, *DPP-4is* dipeptidyl peptidase-4 inhibitors, *GLP-1* receptor agonists, glucagon-like peptide-1, *SGLT2is* sodium-glucose cotransporter 2 inhibitors, *α-GI* alpha-glucosidase inhibitor, *PDEis* phosphodiesterase inhibitors, *ACEis* angiotensin-converting enzyme inhibitors, *ARBs* angiotensin II receptor blockers, *ARNI* angiotensin receptor-neprilysin inhibitor, *V2-Receptor blockers* vasopressin V2 receptor blockers.

**Table 4:** Logistic regression analysis of factors associated with the implementation of revascularization in patients with type 2 diabetes and lower extremity artery disease.

		Odds ratio	95% CI	*P* value
Sex	Male	Reference	–	
	Female	0.46	0.36–0.60	< 0.001
Age/10years		1.25	1.08–1.45	0.002
BMI (kg/m^2^)	≥ 18.5	Reference	–	
	< 18.5	2.00	1.25–3.18	0.004
HbA1c (%)	≥ 7.0	Reference	–	
	< 7.0	0.66	0.52–0.82	< 0.001
eGFR (mL/min/1.73m^2^)	≥ 60	Reference	–	
	30 to <60	1.26	1.04–1.54	0.02
	< 30	2.21	1.48–3.29	< 0.001
Diastolic blood pressure (mmHg)	≥ 80	Reference	–	
	< 80	1.48	1.22–1.79	< 0.001
Systolic blood pressure (mmHg)	≥ 130	Reference	–	
	< 130	0.91	0.73–1.15	0.426
HDL-C (mg/dL)	< 40	Reference	–	
	≥ 40	0.96	0.71–1.31	0.81
LDL-C (mg/dL)	≥ 120	Reference	–	
	< 120	0.90	0.75–1.07	0.23
TG (mg/dL)	≥ 150	Reference	–	
	< 150	0.77	0.65–0.92	0.004
Smoking history	No	Reference	–	
	Yes	1.98	1.63–2.40	< 0.001
Drinking history	Every day	Reference	–	
	Sometimes	1.20	0.98–1.47	0.081
	Rarely	1.35	1.06–1.73	0.015
	No	1.26	0.87–1.81	0.224
Anti-diabetic agent use				
DPP-4is		0.98	0.79–1.21	0.839
Glinides		–	–	–
GLP-1 receptor agonists		0.56	0.37–0.85	0.006
Imeglimin		1.07	0.33–3.49	0.915
Insulin		1.01	0.82–1.25	0.909
Metformin		0.72	0.58–0.90	0.004
SGLT2is		0.52	0.42–0.66	< 0.001
Sulfonylureas		1.21	0.95–1.55	0.122
Thiazolidinediones		–	–	–
α-GIs		1.03	0.80–1.32	0.828
Antiplatelet drug use				
Aspirin		1.40	1.14–1.72	0.001
P2Y12is		2.25	1.83–2.76	< 0.001
PDEis		4.34	3.56–5.28	< 0.001
Antihypertensive drug use				
ACEis		0.88	0.67–1.16	0.368
Aldosterone receptor blocker		1.21	0.92–1.60	0.166
ARBs		1.39	1.14–1.71	0.001
ARNI		1.25	0.82–1.92	0.292
Calcium channel blockers		1.97	1.55–2.49	< 0.001
Loop diuretics		1.09	0.86–1.39	0.466
Thiazide diuretics		1.06	0.83–1.35	0.641
V2-Receptor blockers		1.11	0.70–1.75	0.667
α-Receptor blockers		1.53	1.17–2.00	0.002
β-Receptor blockers		1.54	1.28–1.86	< 0.001
Cholesterol-lowering drug use				
Ezetimibe		0.72	0.54–0.95	0.02
Eicosapentaenoic acid		0.88	0.67–1.15	0.354
Fibrates		0.68	0.51–0.92	0.012
PCSK9is		–	–	–
Statins		0.89	0.73–1.07	0.217

The data shows the results of the multivariate analysis. Glinides, thiazolidinediones, and PCSK9is were excluded from the univariate analysis.

*BMI* body mass index, *CI* confidence interval, *HbA1c* hemoglobin A1c, *eGFR* estimated glomerular filtration rate, *HDL-C* high-density lipoprotein cholesterol, *LDL-C* low-density lipoprotein cholesterol, *TG* triglycerides, *CKD* chronic kidney disease, *DPP-4is* dipeptidyl peptidase-4 inhibitors, *GLP-1* receptor agonists, glucagon-like peptide-1, *SGLT2is* sodium-glucose cotransporter 2 inhibitors, *α-GI* alpha-glucosidase inhibitor, *PDEis* phosphodiesterase inhibitors, *ACEis* angiotensin-converting enzyme inhibitors, *ARBs* angiotensin II receptor blockers, *ARNI* angiotensin receptor-neprilysin inhibitor, *V2-Receptor blockers* vasopressin V2 receptor blockers.

## Discussion

Our study indicates that SGLT2is, metformin, and fibrates may contribute to the attenuation of LEAD severity in individuals with T2D. This trend was consistently observed even when the analysis was confined to patients with T2D who had been diagnosed with LEAD. Both SGLT2is and metformin, which are foundational therapies for T2D, exhibit distinct, yet complementary mechanisms for mitigating atherosclerosis risk. SGLT2is primarily lower plasma glucose levels by promoting urinary glucose excretion and confer additional cardiovascular benefits, including weight reduction, blood pressure lowering, improved lipid profiles, and anti-inflammatory effects^28–30^. These multifaceted actions are pivotal for the prevention of atherosclerosis, particularly in patients with concurrent obesity or hypertension. Conversely, metformin reduces glucose levels by inhibiting hepatic gluconeogenesis and enhancing insulin sensitivity^31,32^. Through its activation of adenosine monophosphate-activated protein kinase (AMPK), metformin exerts anti-inflammatory and endothelial-protective effects^33–35^ while also improving lipid metabolism and diminishing thrombogenic potential. Unlike SGLT2is, metformin does not directly influence body weight or blood pressure but delivers substantial metabolic and vascular benefits^36–38^. In this study, insulin use was significantly associated with a higher OR for severe LEAD. This finding may be attributed to insulin being typically prescribed to patients with a longer duration of T2D. Longer diabetes duration is often associated with more advanced atherosclerosis and increased vascular complications, including LEAD. Furthermore, insulin-treated patients often have a history of poor glycemic control, which may contribute to progressive vascular damage. Although the observed association is statistically significant, the underlying mechanisms require further investigation to clarify the relationship between insulin use and LEAD progression. Numerous studies have highlighted the pleiotropic effects of fibrates, including the stabilization of atheromatous plaques, anti-inflammatory properties, and lipid-lowering effects^39,40^. These multifaceted actions may play a significant role in mitigating the progression of LEAD. In the present study, the use of pemafibrate was associated with a reduced OR for Revasc in patients with LEAD. Pemafibrate is a tissue-specific PPARα agonist, classified as a selective PPAR modulator alpha (SPPARMα), that significantly lowers the risk of severe LEAD. This effect is attributed to two key factors: SPPARMα is more effective than conventional fibrates in improving TG, HDL-C, non-HDL-C, and very low-density lipoprotein levels^41^; and pemafibrate has been shown to induce favorable modifications in the size and composition of atherogenic lipoproteins^42^. This makes pemafibrate a promising therapeutic option for lipid management in the prevention of LEAD progression in patients with T2D. Additionally, ezetimibe significantly reduced the OR for Revasc in patients with T2D and LEAD. Notably, 93.9% of patients who received ezetimibe had a history of previous statin therapy, suggesting that the observed anti-atherosclerotic effect may result from a synergistic interaction between ezetimibe and statins. This combination may enhance the efficacy of cholesterol control, especially in patients at high cardiovascular risk. In contrast, other medications, such as certain antihypertensive or antiplatelet agents, do not offer comparable vascular protection or glycemic control, which may limit their effectiveness in preventing LEAD progression.

Meta-analyses of previous studies have reported that a 1% increase in HbA1c levels is associated with a 15% increase in the incidence of macrovascular complications in patients with type 1 diabetes and an 18% increase in those with T2D^43^. Intensified blood glucose control therapy has been shown to reduce the risk of myocardial infarction and lower-limb amputations^44,45^. However, the Japanese LEAD guidelines note that there is insufficient evidence to suggest that blood glucose control provides preventive benefits. This study provides new insights into this regard.

In previous studies, risk factors for CLTI have been assessed, with factors such as advanced age, renal dysfunction, smoking, and low BMI commonly reported as significant risk factors^46–56^. Recent research has also identified frailty and maintenance dialysis^57,58^ as important risk factors for CLTI, which aligns with the findings of the present study. The Appropriate Blood Pressure Control in Diabetes study (ABCD study)^59^, which included 950 patients with diabetes, demonstrated that aggressive blood pressure control significantly reduced the incidence of cardiovascular events compared with placebo without exacerbating lower limb symptoms. Conversely, the Antihypertensive and Lipid-Lowering treatment to prevent Heart Attack Trial (ALLHAT)^60^ showed a U-shaped correlation between sBp and outcomes, with optimal levels between 120 and 129 mmHg. In the current study, among 67,693 patients with T2D whose sBP was less than 130 mmHg, 37.6% had an sBP of less than 120 mmHg, which may explain the lack of significant findings in the OR. In contrast, a dBP of less than 60 mmHg was observed in 4.0% of the 98,062 patients, which yielded a significantly higher OR, suggesting that maintaining a higher dBP may be beneficial for preserving blood flow to the lower limbs. It is speculated that the high OR of antihypertensive and antiplatelet drugs in this study was due to their administration in high-risk patients, leading to a possible reversal of causality. The lack of significance observed for statins may be attributed to the fact that a substantial percentage of patients with LEAD in this study were already receiving statin therapy, which is typically prescribed more actively to patients with atherosclerosis. Finally, the relationship between lipid-related parameters-namely, LDL-C, HDL-C, and TG levels, and severe LEAD was examined. High LDL-C, high TG, and low HDL-C levels have been identified as risk factors for the worsening of LEAD^5,61^. Previous studies conducted in Japan have also shown a close association between these lipid abnormalities and the concomitant occurrence of LEAD^62,63^. However, in the current study, LDL-C and HDL levels, the cutoff values of which were set based on current guidelines, did not show significance, whereas TG levels of less than 150 mg/dL were associated with a decreased OR. Patients with high LDL-C and low HDL-C levels require more aggressive statin or fibrate treatment. Moreover, LDL-C and HDL-C levels were lower in patients with significantly impaired renal function, suggesting a potential influence of various patient characteristics. Nevertheless, further investigation is warranted because the reasons for this could not be clearly elucidated. In contrast, when TG levels were within the cutoff value, the OR of severe LEAD was significantly reduced in patients with LEAD. Oh et al.^64^ also reported that Intima-Media Thickness was significantly decreased with a TG level of less than 150 mg/dL. We speculate that achieving the cutoff value for TGs in this study may have suppressed atherosclerosis and reduced the risk of severe LEAD. The clinical implications of these findings emphasize the importance of comprehensive management strategies for LEAD, incorporating pharmacological therapies alongside lifestyle modifications, including exercise and smoking cessation. Clinicians should consider the individual characteristics of patients, including age, kidney function, and comorbidities, when selecting medications to manage LEAD and prevent its progression.

This study has a few limitations that must be considered when interpreting the findings. First, the DeSC database mainly covers employment and self-employed insurance, and since the insurance system in Japan changes from 75 years of age and above, much of the data for this age group are missing. Second, the severity of LEAD was assessed by performing Revasc; however, the ABI could not be investigated. Therefore, the exact severity of LEAD could not be determined. This may be subjective to evaluators. Therefore, a prospective study is required to validate our results. Third, severe LEAD was investigated as an outcome in this study. Generally, advanced LEAD is diagnosed as CLTI using the WIfI classification. However, since the claims database does not provide information necessary for the WIfI classification, such as subjective symptoms, it was not possible to define CLTI. Consequently, it was necessary to define the outcome as severe LEAD. Fourth, only 30% of the patients had available laboratory data. As these patients may have unknown factors that were not included in the analysis, their impact on the results cannot be ruled out. Fifth, in individuals near the upper age limit of the database (75 years), the observation period may be relatively short, affecting the assessment of LEAD progression over time. This limitation should be considered when interpreting findings for older patients. Finally, the high ORs observed for antihypertensive and antiplatelet drugs may result from their use in high-risk patients, suggesting a potential reversal of causality. However, it remains unclear whether patients’ condition severity was fully adjusted for in the analysis. Future studies incorporating more comprehensive analyses of patient severity, medication dosage and duration may be required to validate these findings. Previous studies have shown that overlapping risk factors can increase the risk of LEAD onset by 1.5 to 10 times^65^. LEAD is also frequently associated with other atherosclerotic diseases, such as CVD, and is associated with a 1.5- to 2-fold higher risk of cardiovascular events even after adjustment for age^66^. Therefore, comprehensive management of risk factors is essential not only to prevent the development and progression of LEAD, but also to improve overall prognosis. However, LEAD is often underrecognized in clinical practice, and even when diagnosed, its management tends to be inadequate^67–70^. Beyond pharmacological treatment, a comprehensive risk management approach—including lifestyle interventions such as exercise and smoking cessation—should be regarded as a fundamental aspect of LEAD care. These findings support the necessity of individualized patient management in the clinical setting.

In conclusion, the active use of SGLT2is, metformin, and fibrates may be beneficial in preventing LEAD progression. However, these medications are associated with a higher incidence of adverse events, particularly among elderly patients or those with impaired kidney function. Careful individualized patient management is essential to maximize the benefits of these treatments. Future studies further evaluating the factors associated with LEAD may be required to validate these findings and inform guidelines for LEAD prevention and management. Additionally, to establish accurate causal relationships, randomized controlled trials are necessary.

## Materials and methods

### Study design

This retrospective cross-sectional study utilized data from the DeSC database (DeSC Healthcare, Inc., Tokyo, Japan), which includes anonymized health insurance claims collected via a web-based platform (kencom) operated by DeSC Healthcare Inc. The database integrates information from two categories of insurers: corporate health insurance associations covering employees of large Japanese companies and their dependents (< 75 years old), and national health insurance providers for self-employed individuals and others not covered by other insurance (< 75 years old). Health insurance claims data included: patient demographics; diagnoses coded according to the International Classification of Diseases, 10th Revision (ICD-10); medical procedures; and monthly prescription details. The health checkup data encompassed results from annual physical examinations, biomarker assessments, imaging studies (e.g., chest X-rays), and questionnaire responses addressing medical history, comorbidities, medication usage, and lifestyle factors such as smoking. Additional self-reported data, including responses to the Work Productivity and Activity Impairment Questionnaire, were gathered via a web-based system. Data were anonymized through an “opt-out agreement” with users, who were informed of the potential utilization of their data and their right to request deletion. DeSC encrypts all patient data to ensure anonymity for database users. This study analyzed the data collected between April 1, 2014 and October 30, 2023. Prescription records and information on targeted medications were reviewed for the period spanning the diagnosis of LEAD and the subsequent endovascular treatment.

### Ethical considerations

This study was conducted in accordance with the Declaration of Helsinki and the Ethical Guidelines for Medical and Health Research Involving Human Subjects. This study was approved by the Ethical Committee of Musashino University (No. R6-7). Since unlinked, anonymized data were used, the ethics committee confirmed that this study was not subject to compliance with the Ethical Guidelines for Medical and Health Research Involving Human Subjects, Due to the retrospective nature of the study, the Ethical Committee of Musashino University waived the need of obtaining informed consent.

### Study population

**Fig. 1:**
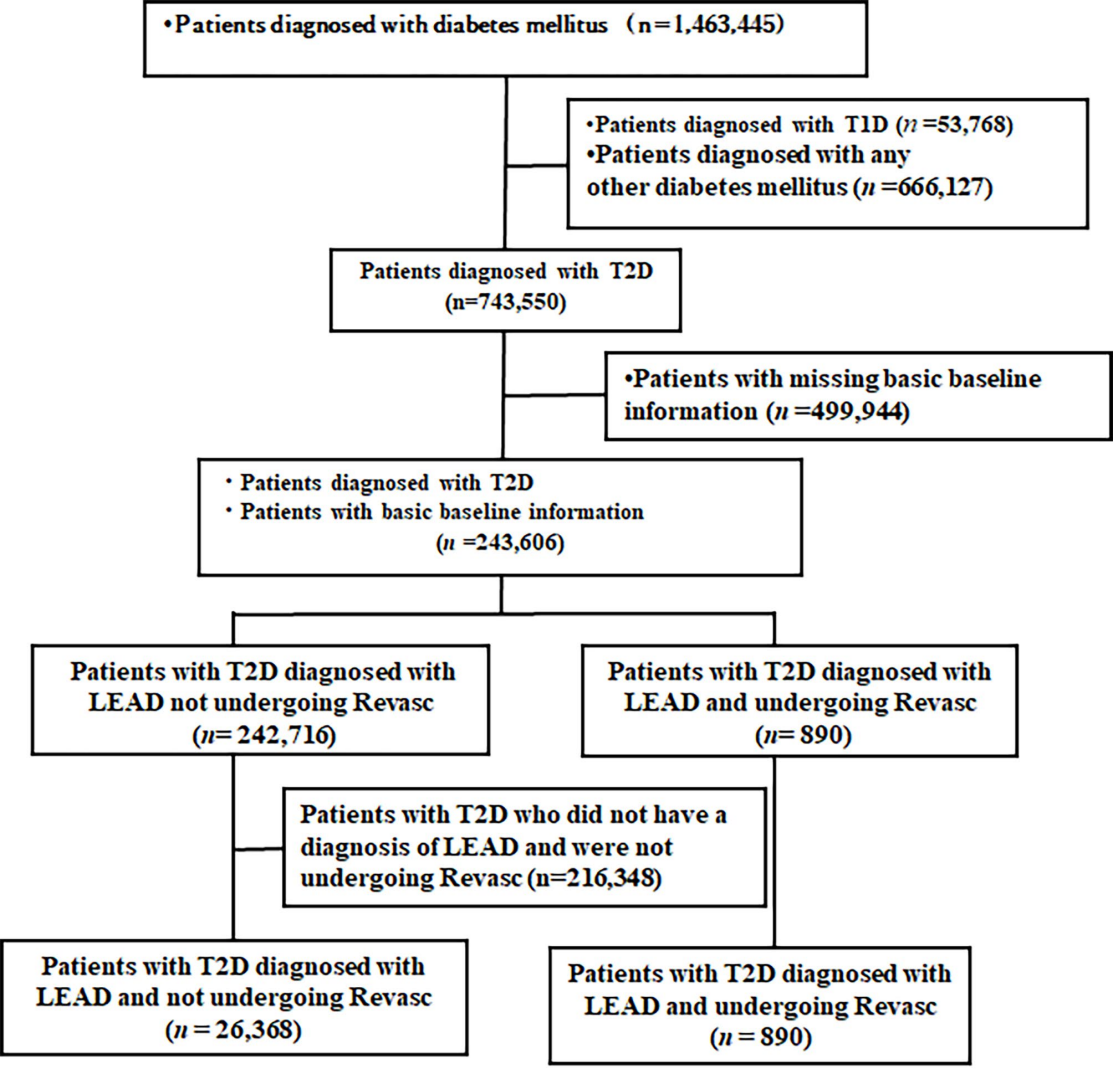
Patient disposition. *LEAD* lower extremity artery disease, *T1D* type 1 diabetes, *T2D* type 2 diabetes, *Revasc* revasculation.

First, the data of 1,463,445 patients with diabetes mellitus (ICD-10 codes: E11–E14) registered between April 1, 2014 and October 30, 2023, in the DeSC claim database were extracted (Fig. 1). Next, patients with T2D were selected based on their most recent diabetes-related ICD-10 code. The following patients were excluded: patients diagnosed with type 1 diabetes (*n* = 53,768), patients diagnosed with any other diabetes mellitus (*n* = 666,127), and patients with missing basic baseline information (*n* = 499,944). Finally, 243,606 patients with T2D were included and subsequently divided into two groups: patients with T2D diagnosed with LEAD undergoing Revasc (*n* = 890) and patients with T2D not undergoing Revasc (*n* = 242,716). In the subgroup analysis, we focused exclusively on patients with T2D who were diagnosed with LEAD. Hence, patients with T2D who did not have a diagnosis of LEAD (*n* = 216,348) were excluded. Finally, 27,258 patients with T2D who were diagnosed with LEAD were included and subsequently divided into two groups: patients with T2D diagnosed with LEAD and undergoing Revasc (*n*=]890) and patients with T2D diagnosed with LEAD and not undergoing Revasc (*n* = 26,368).

### Identification of complications, use of concomitant drugs, and revasc events

Complications were identified based on ICD-10 codes, assigned up to the time of Revasc implementation for patients who underwent Revasc, and throughout the observation period for those who did not. Concomitant medications were investigated using the same approach. Revasc events were defined as medical procedures classified as Revasc that were performed after the assignment of the ICD-10 code for LEAD. Revasc encompassed medical procedures such as bypass surgery and EVT. The severity of LEAD and its complications were assessed following the criteria established in a prior study by Felix et al.^71^, which defined the severity of diabetic complications. To ensure the accuracy of database coding for complications and procedures, we relied on the adapted Diabetes Complications Severity Index (DCSI), which uses ICD-10 codes to identify and classify diabetes-related complications. This method has been validated in previous studies^72^, which demonstrated the reliability of the DCSI in assessing complications in patients with diabetes. The DeSC database also undergoes regular audits and validation checks to verify the accuracy of clinical event codes, further ensuring the reliability of the data used in this study. Using these validated tools and processes supports the credibility of these findings and minimizes potential coding errors. The Revasc procedures examined in this study are detailed in Supplementary Table S1.

### Outcomes

Revasc is recommended as a treatment option in patients with T2D and LEAD who present with ischemic rest pain or exercise-induced claudication if symptoms are inadequately managed by pharmacotherapy or exercise therapy^73^. In the present study, severe LEAD was defined as a diagnosis of LEAD for which Revasc was performed.

### Patient characteristics

Age, sex, body mass index (BMI), and laboratory data were identified using data recorded in the claim records closest to the start of the observation period. Being underweight was defined by a BMI of less than 18.5 kg/m^2^. Laboratory data were collected to determine the estimated glomerular filtration rate (eGFR), hemoglobin A1c (HbA1c), high-density lipoprotein cholesterol (HDL-C), low-density lipoprotein cholesterol (LDL-C), triglyceride (TG), diastolic blood pressure (dBp), and systolic blood pressure (sBp).

### Statistical analysis

Normally distributed data (age, BMI, eGFR, HbA1c, HDL-C, LDL-C, TG, dBP, and sBp) are expressed as means ± standard deviations and were analyzed using an unpaired *t*-test. Categorical variables are expressed as absolute numbers or percentages and analyzed using the χ^2^ or Fisher’s exact test. The cutoffs were set at less than 7.0% for HbA1c; 40 mg/dL or more for HDL; less than 120 mg/dL for LDL; less than 150 mg/dL for TGs; less than 30 mL/min/1.73 m^2^, and between 30 and 60 mL/min/1.73 m^2^ for eGFR; less than 80 mmHg for dBP; and less than 130 mmHG for sBp, according to the guidelines^29^. Variables significantly associated with severe LEAD were identified using univariate logistic regression. Those with *P* < 0.2 were included in the multivariate model. To reduce potential multicollinearity, although formal diagnostics (e.g., variance inflation factors) were not performed, variable selection was guided by both statistical significance and clinical relevance.　Risks are expressed as odds ratios (ORs) with their corresponding 95% confidence intervals (CIs). This study excluded patients with missing baseline data from the analysis. As a result, there was no need to handle missing data within the analysis population. We used forced entry methods to include clinically relevant variables, such as patient background and known risk factors for LEAD progression. Forced entry ensures that all key factors are included, regardless of statistical significance. Regarding multicollinearity, clinically important factors were prioritized, and comorbidities were excluded due to suspected high collinearity with concomitant medications. Regarding the criteria for exclusion of variables, particularly comorbidities, we considered the potential for multicollinearity between diagnosis codes and concomitant medications. In our dataset, it was difficult to distinguish between individuals who were diagnosed but not treated (e.g., due to mild or subclinical disease), and those who received a diagnosis for administrative or screening purposes without clinical confirmation. Since pharmacological treatment often follows clinical diagnosis, we assumed that medication use could serve as a more reliable proxy for clinically significant disease than diagnosis codes alone. Including both medication and diagnosis variables in the same model could lead to multicollinearity and obscure true associations. Therefore, we prioritized the inclusion of medication variables to minimize potential misclassification and enhance the robustness of the model.　All statistical analyses were performed using IBM SPSS Statistics for Windows version 23.0 (IBM Corp., Armonk, NY, USA). Statistical significance was set at a *P*-value of less than 0.05.

## Electronic supplementary material

Below is the link to the electronic supplementary material.

Supplementary Material 1

## Data Availability

The data that support the findings of this study are available from DeSC database but restrictions apply to the availability of these data, which were used under licence for the current study, and so are not publicly available. Data are, however, available from the corresponding author, Takeshi Horii (t-horii@musashino-u.ac.jp), upon reasonable request and with permission from DeSC Healthcare, Inc.
