# Supplementary material for: Factors associated with severe lower extremity artery disease in type 2 diabetes based on a large scale claims database in Japan
Source: Sci Rep. 2025 Jun 3;15:19358. doi: 10.1038/s41598-025-03797-9 (PMC12134259; doi:10.1038/s41598-025-03797-9)
Supplement: Supplementary file 1 — Supplementary Material 1 [file 41598_2025_3797_MOESM1_ESM.pdf]

Table S1, A list of medical procedures covered as revascularization.

	Name of medical procedure
Endovascular therapy	• Arterial embolectomy (other (open surgery)<br>• Open stent-graft placement (descending aorta)<br>• Percutaneous intravascular foreign body removal<br>• Percutaneous lower limb arterioplasty<br>• Thrombosis endarterectomy (extending to aorta)<br>• Thrombosis endarterectomy (internal carotid artery)<br>• Thrombosis endarterectomy (other)<br>• Vasodilation-thrombectomy of extremity
Bypass surgery	• blood vessel grafting, bypass grafting (artery-to-artery bypass with autologous blood vessels for femoral artery occlusion)<br>• blood vessel grafting, bypass grafting (lower leg, foot artery)<br>• blood vessel grafting, bypass grafting (other artery)<br>• blood vessel grafting, bypass grafting (popliteal artery)<br>• vascular anastomosis of extremity

Table S2. Logistic regression analysis of factors associated with the implementation of revascularization in patients with type 2 diabetes / component-specific analysis of SGLT2 Inhibitors and Fibrate Drugs

	Odds Ratio	95%CI	P value
SGLT2is			
Canagliflozin	0.75	0.54-1.06	0.10
Dapagliflozin	0.45	0.33-0.61	<0.001
Empagliflozin	0.64	0.49-0.82	<0.001
Ipragliflozin	0.78	0.57-1.06	0.11
Luseogliflozin	0.88	0.57-1.34	0.54
Tofogliflozin	0.72	0.46-1.12	0.15
Fibrates			
Bezafibrate	0.86	0.61-1.22	0.39
Clinofibrate	1.50	0.04-52.13	0.82
Fenofibrate	0.93	0.65-1.33	0.69
Pemafibrate	0.39	0.24-0.65	<0.001

The data show the results of the multivariate analysis. SGLT2is, sodium-glucose cotransporter 2 inhibitors. Clofibrate were excluded from the univariate analysis.

Table S3. Logistic regression analysis of factors associated with the implementation of revascularization in patients with type 2 diabetes and lower extremity artery disease / component-specific analysis of SGLT2 Inhibitors and Fibrate Drugs

	Odds Ratio	95%CI	P value
SGLT2is			
Canagliflozin	0.81	0.57-1.15	0.23
Dapagliflozin	0.48	0.35-0.66	<0.001
Empagliflozin	0.62	0.47-0.81	<0.001
Ipragliflozin	0.75	0.54-1.03	0.08
Luseogliflozin	0.82	0.53-1.28	0.39
Tofogliflozin	0.71	0.45-1.12	0.14
Fibrates			
Bezafibrate	0.91	0.63-1.31	0.62
Fenofibrate	0.95	0.65-1.39	0.79
Pemafibrate	0.34	0.20-0.58	<0.001

The data show the results of the multivariate analysis. SGLT2is, sodium-glucose cotransporter 2 inhibitors. Clinofibrate and Clofibrate were excluded from the univariate analysis.
